# Cost-effectiveness of mandatory bicycle helmet use to prevent traumatic brain injuries and death

**DOI:** 10.1186/s12889-020-08544-5

**Published:** 2020-03-30

**Authors:** Rick Reuvers, Eelco A. B. Over, Anita W. M. Suijkerbuijk, Johan J. Polder, G. Ardine de Wit, Paul F. van Gils

**Affiliations:** 1 National Institute for Public Health and the Environment, RIVM, Postbus 1, Bilthoven, 3720 BA the Netherlands; 2 Tranzo, School of Social and Behavioral Sciences, Tilburg University, Tilburg, The Netherlands; 3 Julius Center for Health Sciences and Primary Care, University Medical Center Utrecht, Utrecht University, Utrecht, the Netherlands

**Keywords:** Bicycle helmet use, Cost-effectiveness, Traumatic brain injury, Economic evaluation, The Netherlands

## Abstract

**Background:**

Traumatic brain injury (TBI) is the main cause of mortality and severe morbidity in cyclists admitted to Dutch emergency departments (EDs). Although the use of bicycle helmets is an effective way of preventing TBI, this is uncommon in the Netherlands. An option to increase its use is through a legal enforcement. However, little is known about the cost-effectiveness of such mandatory use of helmets in the Dutch context.

The current study aimed to assess the cost-effectiveness of a law that enforces helmet use to reduce TBI and TBI-related mortality.

**Methods:**

The cost-effectiveness was estimated through decision tree modelling. In this study, wearing bicycle helmets enforced by law was compared with the current situation of infrequent voluntary helmet use. The total Dutch cycling population, consisting of 13.5 million people, was included in the model. Model data and parameters were obtained from Statistics Netherlands, the National Road Traffic Database, Dutch Injury Surveillance System, and literature. Effects included were numbers of TBI, death, and disability-adjusted life years (DALY). Costs included were healthcare costs, costs of productivity losses, and helmet costs. Sensitivity analysis was performed to assess which parameter had the largest influence on the incremental cost-effectiveness ratio (ICER).

**Results:**

The intervention would lead to an estimated reduction of 2942 cases of TBI and 46 deaths.

Overall, the incremental costs per 1) death averted, 2) per TBI averted, and 3) per DALY averted were estimated at 1) € 2,002,766, 2) € 31,028 and 3) € 28,465, respectively. Most favorable were the incremental costs per DALY in the 65+ age group: € 17,775.

**Conclusions:**

The overall costs per DALY averted surpassed the Dutch willingness to pay threshold value of € 20,000 for cost-effectiveness of preventive interventions. However, the cost per DALY averted for the elderly was below this threshold, indicating that in this age group largest effects can be reached. If the price of a helmet would reduce by 20%, which is non-hypothetical in a situation of large-scale purchases and use of these helmets, the introduction of this regulation would result in an intervention that is almost cost-effective in all age groups.

## Background

In the Netherlands, cycling is a common mode of transportation and bicycle trips account for up to 28% of all transfers made [1]. Dutch people cycle a mean daily distance of 2.9 km and over 40% of inhabitants cycle at least once a day, the highest level of any country in the European Union [2, 3]. Although the level of road safety in the Netherlands is high, roughly 78,400 injuries per year are treated at an emergency department (ED), of which 13,300 (17%) result in hospital admissions [4]. Even though the total number of bicycle related fatalities declined between 1996 (239) and 2016 (189), the number of bicycle related deaths remained at a high level with an average number of 189 casualties in the last 5 years [5]. In 1996, 20% of all traffic deaths were bicycle related, increasing to 30% in 2016 [5].

Traumatic brain injury (TBI) is the main cause of severe morbidity and mortality after an accident involving bicyclists. More specifically, 32% out of all hospitalized cyclists with severe injury has a head or brain injury [1, 4, 6]. The incidence of bicycle related TBI treated at the ED has increased with 54% between 1998 and 2012, while the overall incidence of bicycle related injuries treated at the ED remained relatively stable in that period. In 2012, bicycle related TBI was treated 43 times per 100,000 persons in the ED and resulted in subsequent hospitalization in 64% of the TBI cases [6].

Most survivors of this form of injury remain impaired for life. TBI has been associated with a decline in cognitive capacity, long lasting physical disability and handicap, and the development of mental illness [7–9]. The consequences of TBI often prohibit survivors from returning to full employment, reduce their quality of life, and have been linked to an increased risk of suicide [10–13]. Scholten et al. valued the mean healthcare costs and costs due to productivity loss resulting from bicycle related TBI at € 19,620 per case for the Netherlands, of which € 4940 healthcare costs and € 14,680 productivity losses per case. Total costs were estimated at 74.5 million euros in 2012 [6].

About 75% of all bicycle-related head injuries are caused by single-bicycle accidents, i.e. accidents without any motorized vehicles involved. In most cases this involves falls or collisions with an obstacle [14]. Polinder et al. (2016) identified these injuries as a priority area for prevention [15]. The use of bicycle helmets has been found to be an effective measure of preventing head and brain injuries, especially in the case of these single-bicycle accidents [16–19]. It is associated with a 51% reduction in odds for head injury, according to the most recent and comprehensive systematic review on the subject [18]. Several countries have introduced legislation, which enforces the use of helmets. Examples are Spain, Sweden, Australia, New Zealand, certain states in Canada, and the United States [17]. However, many countries only require children to wear helmets. A review of population-based studies that compare injury data before and after the introduction of legislation that enforces use of bicycle helmets found decreases in head injuries and mortality in certain parts of Canada and the United States [17]. However, some other similar studies found only a small or no obvious effect of legislation on number of injuries in New Zealand and certain parts of Australia and Canada [20, 21].

Bicycle helmets are not mandatory in the Netherlands and are generally only used by young children, mountain bikers and racing cyclist [22]. According to a survey commissioned by the Royal Dutch Touring Club, 73% of adults and 84% of children under 17 never wear a helmet [23]. Although a considerable amount of research into the efficacy of both the bicycle helmet and legislation that enforces its use has been performed, the cost-effectiveness of such a law is unknown in the Dutch context. The current research aims to explore the cost-effectiveness of mandatory helmet use by comparing costs and benefits of legislation with the costs and benefits of the existing situation, i.e. voluntary helmet use in a small part of all cyclists. This study can support decision-making concerning a legalization to prevent bicycle related TBI and death [24, 25].

## Methods

### Study design

The cost-effectiveness of legislation that enforces the use of bicycle helmets was assessed through a decision-tree model based on parameters obtained from the literature and several databases. According to the Dutch guideline for economic evaluations, a societal perspective was used, implying that not only healthcare costs but also non-healthcare costs such as cost of lost productivity, were included [26].

The model had a lifetime horizon and compared the cost and effects of the status quo, voluntary helmet use, with the cost and effects of the intervention, mandatory helmet use for all cyclists regardless of age.

In this economic evaluation, behavioral changes as a result of the compulsory wearing of a helmet were not taken into account because conclusive evidence on such behavioral changes were not available.

### Study population

The model contained a cohort that consists of all Dutch cyclists that use a non-racing or non-mountain bike, i.e. users of a utility bicycle. It was assumed that users of racing and mountain bikes are also users of the utility bicycle. This type of bicycle is most common for daily use in the Netherlands and will hereafter simply be referred to as ‘bicycle‘. The total number of cyclists was retrieved from Statistics Netherlands [27]. Data on active traffic participants were available for specific age groups in the year 2016 and were corrected for population growth in 2017 (base year) in the corresponding age groups [28]. The resulting total number of approximately 13.5 million cyclists match the raw estimate given by the National Road Traffic Database [29]. Cyclists were divided into three age groups: up to 15 years, between 15 and 65 years, and 65 years and older.

### Intervention and comparator

The intervention under analysis is a hypothetical law imposing everyone in the Netherlands to wear a helmet when riding a utility bicycle. The comparator is the current situation of only voluntary helmet use.

#### Model parameters

- *TBI-related disease burden without a helmet law, the current situation*

Age specific estimates of bicycle-related TBI incidence and mortality were obtained from the Dutch Injury Surveillance System (LIS) and the mortality statistics of Statistics Netherlands (Table 1) [30, 31]. The LIS is based upon 13 geographically distributed Emergency Departments (EDs) in the Netherlands, resulting in a representative 12% sample of injury-related ED visits. Age specific estimates on DALYs following bicycle related TBI were obtained from the Dutch Burden of Injury Model [32, 33]. These were specified as years lived with disability (YLD) for people that survived bicycle related TBI and years of life lost (YLL) for people that died due to bicycle related TBI. The disability weights for temporary and hospitalized brain injury were 0.090 and 0.241, respectively [34].

**Table 1:** Model parameters, not specified by age group

Model parameters	Value (registered cases TBI on ED)	Source
Incidence of TBI (number of cases)	8016	Dutch Injury Surveillance System
TBI related mortality (number of cases)	124	CBS cause of death statistics
Years Lived with Disability after TBI (mean value)	6649	Dutch Burden of Injury Model
Years of life lost following TBI (mean value)	2089	Dutch Burden of Injury Model
Disability-adjusted life years (sum of YLL and YLD)	8738	Dutch Burden of Injury Model
Number of cyclists	13,468,742	Computed with data from CBS Statline
Helmet efficacy (RR: TBI with helmet vs. without helmet)	0.583	Computed with data from existing literature
Mean current helmet use rate	2%	Dutch Injury Surveillance System
Mean helmet use rate following legislation	88%	Computed with data from existing literature
Risk of TBI when helmeted	0.000347	Model outcome
Risk of TBI when unhelmeted	0.000595	Model outcome
Risk of death for TBI victim	0.015493	Model outcome
Mean medical costs per person with TBI	€ 4335	Model outcome
Incidence of work absenteeism^*^ after TBI (number of cases)	2820	Model outcome
Mean work absenteeism costs* per person	€ 11,252	Model outcome
Mean working-days absent	36	Model outcome
Mean productivity loss due to mortality^*^	€ 12,054	Model outcome
Friction period	101 days	Guideline [26]
Mean number of working hours per week	31 h	CBS Statline
Net labor participation	75.80%	CBS Statline
Mean wage per hour	€ 35.55	Manual for Costing Research, corrected for inflation
Mean helmet costs per year	€ 10	Estimate (see text)

Work absenteeism costs and productivity loss were calculated with the friction-cost method

*Only for age group 15–65 years

All data used from the Dutch Injury Surveillance System, Dutch Burden of injury Model, and national mortality statistics relate specifically to users of utility bicycles in 2017. The annual probability of having TBI was calculated by dividing the incidence of TBI by the total number of cyclist within each age group (≤14, 15–64, ≥65 year). Because of the very low voluntary helmet use in the country, it was assumed that none of the cases with TBI had worn a helmet. The probability of mortality due to bicycle related TBI was calculated by dividing the mortality by the incidence of TBI within each age group.

b. *Helmet use*

An estimate on bicycle helmet efficacy was obtained from a recent and comprehensive review and meta-analysis [18]. As this estimate is presented as an odds ratio (OR), it was recalculated into a relative risk with the following equation: RR = OR (1− P_ref_) + (P_ref_ ∗OR), where P_ref_ stands for the prevalence of TBI in the group not wearing a helmet [35].

The overall helmet-wearing rate of all injured cyclists that were registered in the Dutch Injury Surveillance System was obtained (2%). This rate was also taken as the helmet-wearing rate in the general population. Estimates on compliance to mandatory bicycle helmet use in other countries were obtained by combining data from literature. Helmet wearing rates following introduction of legislation in New Zealand, Canada, the United States, and Australia were used [21, 36–39]. In case of several estimates being available for a single country, the mean helmet-wearing rate for that country was calculated. The mean helmet-wearing rate over all countries was added to the helmet-wearing rate in the Netherlands following the introduction of the mandatory helmet for moped drivers in 1975 and the mean of those two variables was considered the projected helmet-wearing rate.

c. *TBI-related disease burden following legislation*

Calculations were as follows:

- Probability of having TBI for helmet wearers was calculated by multiplying the baseline probability of acquiring TBI as a bicyclist with the relative risk (RR) of TBI for helmet wearers.
- Incidence of TBI for helmet wearers was obtained by multiplying the projected helmet-wearing rate with the total number of cyclists for a specific age group. This group of helmet wearers was then multiplied with the probability of having TBI for helmet wearers.
- Incidence of TBI for non-helmet wearers was obtained by multiplying the projected group of non-helmet wearers with the probability of having TBI for non-helmet wearers.
- Mortality was calculated by multiplying the bicycle related TBI incidence with the probability of mortality due to bicycle related TBI.
- YLD was calculated by multiplying the YLD in the situation without a helmet law with the ratio of the incidence of TBI in the situation with the helmet law to the incidence of TBI in the situation without the helmet law.
- YLL was calculated by multiplying the YLL in the situation without a helmet law with the ratio of the mortality in the situation with the helmet law to the mortality in the situation without mandatory helmet use.

#### Costs

The types of costs that were included in the model were healthcare costs, costs due to lost productivity for both people that survived TBI and for people that died from TBI, and the annual cost of purchasing helmets. All costs were expressed in 2017 euros.

- *Healthcare costs*

Treatment costs of bicycle related TBI were obtained from the Dutch Burden of Injury Model and included general practitioner care, ambulance transport, hospital, physiotherapy, home care and domestic help, nursing homes, and rehabilitation care. These costs occurred within the first year after the injury and were assumed an acceptable representation of the total treatment costs. No data are available for the health care costs after year 1. Total treatment costs for survivors under the helmet law condition were calculated by multiplying the projected incidence of TBI with the mean treatment costs per TBI.

b. *Productivity losses*

The loss of productivity of people that survived TBI were also obtained from the Dutch Burden of Injury Model and were calculated using the friction-cost method, as advised by the Dutch guidelines for health economic evaluation [26]. Only people of working age were eligible to incur this type of cost. The friction cost period was converted into working hours and multiplied by the mean productivity costs per hour for a working person in 2017 [26, 40–42]. Total productivity losses for survivors under the helmet law condition are obtained by multiplying the projected incidence of TBI with the mean productivity losses for survivors. Total productivity losses for people that have died were obtained by multiplying the projected mortality with the mean productivity losses for this group.

c. *Helmet costs*

The mean bicycle helmet price was estimated in the same way as was done in German cost-benefit-research [43]. The cost of the cheapest helmet of good quality in the most recent review of ‘Stiftung Warentest’ (consumer product tests) was used and was indexed to Euros 2017 [44–46]. The German figures were used because of the large market share and the most recent data.

The mean recommended retail price for the twelve best-selling helmets on www.amazon.de was also used. The mean of these two prices was taken and corrected for Dutch price levels [47]. Everyone from the age of fifteen onwards was assumed to wear a helmet for adults. Helmets were assumed to be replaced every 5 years, as this is a period that is cited frequently in other bicycle helmet research [43]. Yearly helmet costs were calculated as one fifth of these total costs. Total helmet costs for the status quo were calculated by multiplying the current percentage of helmet use with the total number of cyclists. Total helmet costs under the helmet law condition were calculated by multiplying the projected helmet-wearing rate with the total number of cyclists for each specific age group, which was then multiplied by the mean helmet price for that age group.

#### Outcomes

Incremental cost-utility ratios (ICERs) were estimated by dividing the difference in costs between the current situation and the implementation of mandatory bicycle helmet use, by the difference in effects between both scenarios. This means that ICERs were estimated for respectively bicycle related TBI, death, and DALYs averted.

#### Sensitivity analyses

One way sensitivity analysis was performed to assess the uncertainty surrounding the following model parameters: incidence of TBI, mortality, YLL, disability adjustments in YLD, number of years lived with a disability, costs of healthcare resources use, costs due to productivity loss, annual helmet costs, current helmet wearing percentage, projected future helmet wearing percentage, efficacy of the helmet, and total number of cyclists. The scores were decreased and increased by 20% for each variable separately, except for the projected helmet-wearing rate. This variable was increased to its maximum value of 100%. Results of the sensitivity analyses were displayed in tornado plots. Annual helmet costs were varied with a factor in the range of 10–200%.

Additionally, the human-capital method was used to calculate productivity losses for people that died due to TBI. The use of this method assumes that productivity is lost for the full duration of absence from work until age of retirement, and hence, that costs of productivity losses stretch out over a much longer period than the friction period only [48].. These costs were calculated by subtracting the mean age of death for the group of working age from the retirement age of 65 years and multiplying this with the mean amount of weeks per year, mean working hours per week, net labor participation rate, and mean productivity costs per hour for a working person in 2017. The same was done for the age group younger than 15 years with the same formula, except that they start with a full 50 years of labor left.

#### Discounting

Effects and costs that occurred after 1 year were discounted in accordance with the Dutch guideline for economic evaluations [26]. YLL and YLD were discounted at a discount rate of 1.5% per year. Having TBI was assumed not to influence the life expectancy for survivors of TBI, as information about age-specific life expectancy for TBI survivors was not available. Therefore, YLD was discounted for the number of years that the mean person within an age group was projected to live. Productivity losses for people that had died because of bicycle related TBI were only discounted in the sensitivity analysis in which the HC-method was used, because the friction method that was used for the base-case analysis does not extend beyond a 1 year period. A discount rate of 4% per year was used for all costs.

## Results

Table 1 shows the values of all variables that were included in the model. The model included 13,468,742 cyclists (see Appendix 1 for age distribution). Implementation of a helmet law was projected to lead to a helmet wearing rate of 88% and helmet wearers were assumed to have a relative risk for TBI of 0.583 (CI95 0.513–0.658).

### Effects

In 2017, 8016 cases of TBI were registered in Dutch EDs among users of utility bicycles (Table 2), of which 124 died. TBI per 100,000 inhabitants was approximately twice as high in people of 65 years and older, compared to other age groups. Mortality per 100,000 inhabitants was also highest in people of 65 years and older with a mortality rate that was fifteen and eight-fold higher than in people under 15 years of age and people between 15 and 65 years, respectively. The total burden of TBI related disease was 11,746 DALYs (2.620 LYL and 9.126 YLD) The burden of disease relative to the incidence of TBI was highest in people under 15 years due to a relatively high number of YLD. The bottom part of Table 2 displays the projections of incidence of TBI and subsequent mortality for cyclists after implementation of a helmet law.

**Table 2:** Yearly bicycle related TBI, subsequent mortality and related disease burden in the Netherlands without a helmet law (top), with a helmet law (middle) and the difference between these scenarios (bottom). Undiscounted figures

Age	TBI (n)	per 100,000	Mortality (n)	per 100,000	YLD(n)	LYL (n)	DALY (n)
Without Law							
< 15	1053	38	5	0.176	2494	351	2845
15–64	4558	41	37	0.332	5408	1432	6840
≥ 65	2405	76	82	2.606	1224	837	2061
Total	8016	47	124	0.727	9126	2620	11,746
With Law							
< 15	667	24	3	0.105	1579	222	1801
15–64	2885	26	23	0.215	3424	906	4330
≥ 65	1523	48	52	1.587	775	530	1305
Total	5075	30	79	0.457	5777	1658	7435
Difference							
< 15	− 386	−14	−2	−0.071	− 915	− 129	− 1044
15–64	− 1673	−15	− 14	−0.117	−1985	− 525	− 2510
≥ 65	− 883	− 28	−30	− 1.019	− 449	− 307	− 756
Total	−2942	− 17	−46	−0.270	− 3349	− 961	−4310

The introduction of a helmet law is projected to lead to a yearly reduction of 2942 cases of TBI and 46 deaths across all age groups (Table 2). The number needed to treat for TBI was 4029 for the overall age group. Overall disease burden was reduced by 4310 DALYs, most of which were averted in people between the age of 15 and 65 years (Table 2).

### Costs

**Fig. 1:**
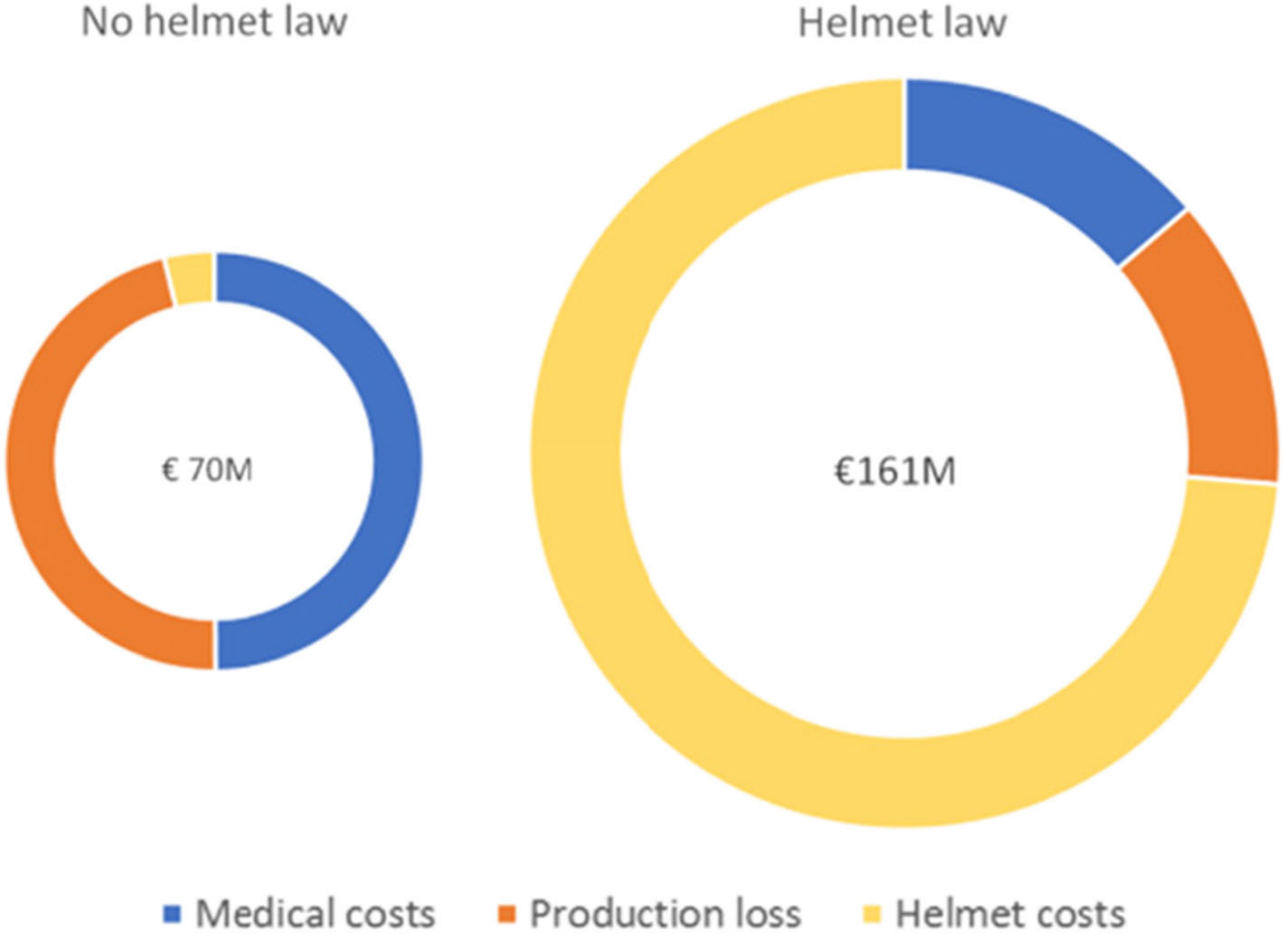
Overview of total costs per scenario and spending per cost-type

The decrease in TBI leads to approximate annual mean savings of 12.8 million euros in medical costs and 11.8 million euros due to averted productivity losses. However, total annual depreciated spending on bicycle helmets rose with approximately 116 million euros, which lead to incremental cost of more than 91 million euros (see Fig. 1).

### Cost-effectiveness

Table 3 shows that there was a large fluctuation in cost-effectiveness between the age groups, with the ICERs for cost per TBI prevented, cost per death prevented, and cost per DALY averted being most favourable for people of 65 years and older, € 17,775 per DALY. Cost per TBI and death prevented were highest for people aged 14 and younger (€ 19,152,803 and € 10,676, 247), while cost per DALY averted was highest in the middle age group (€ 40,519). Overall, the costs per death averted were estimated at € 22,002,76, per TBI averted € 31,028, and per DALY € 28,465. Use of the human-capital approach for calculating the loss of productivity leads to cost-effectiveness estimates being approximately 10% lower for the youngest age group and 15% lower for the middle age group, when compared to the use of the friction-costs method (Appendix 2).

**Table 3:** Overview of incremental cost-effectiveness ratios, friction-costs method. Discounted figures

Age	per averted TBI	per prevented death	per averted DALY
< 15	€ 49,559	€ 10,676,247	€ 30,217
15–64	€ 35,698	€ 4,401,171	€ 31,856
≥65	€ 14,065	€ 410,871	€ 17,775
Total	€ 31,028	€ 2,002,766	€ 28,465

### Sensitivity analyses

**Fig. 2:**
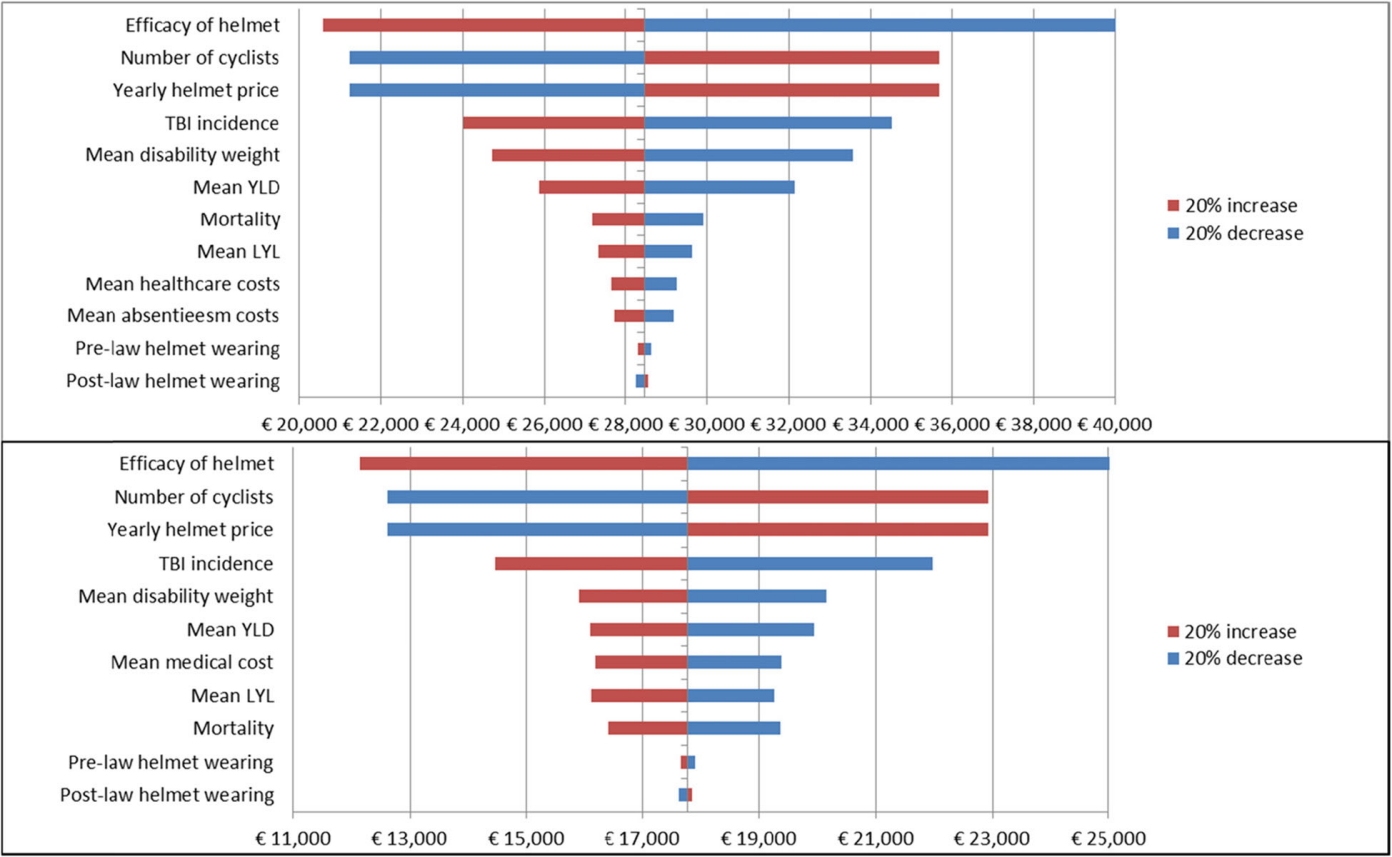
Results of one-way sensitivity analyses for the ICER of cost per DALY averted in the overall group (above) and the age group above 65 years (below)

**Fig. 3:**
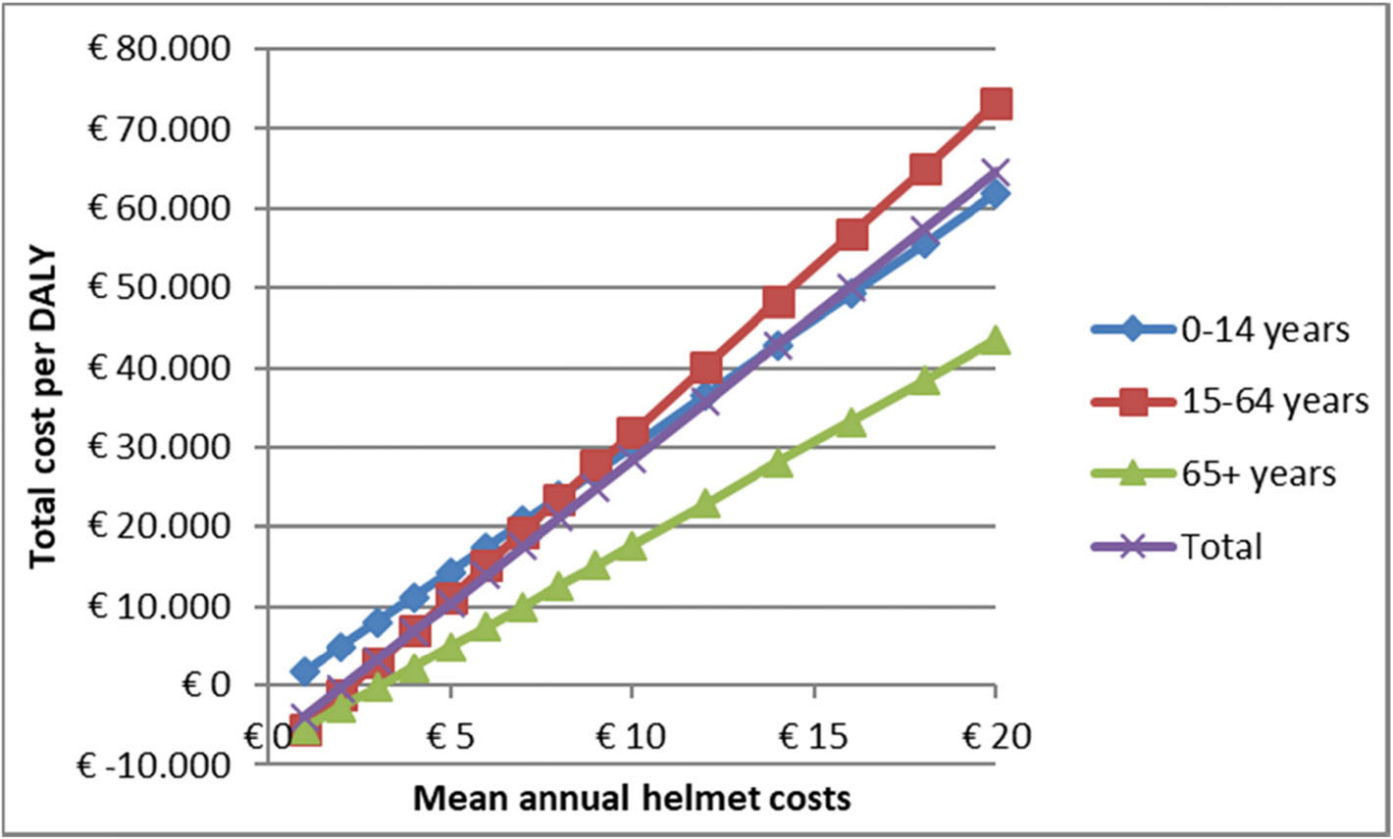
Cost per DALY as a function of annual helmet costs in the range of 10–200% of the standard helmet costs

The sensitivity analyses revealed that the parameter value for helmet efficacy had the largest influence on the ICER. A 20% increase in efficacy lowered the cost per DALY to around € 20,000 and a 20% decrease raised the costs per DALY to around € 42,000 for the overall group (Fig. 2). Total number of cyclists, the annual-helmet costs and incidence of TBI were additional variables that influence results to a large degree. These variables influenced the ICER of the age group of people aged 65 years and older sufficiently for them to possibly surpass the threshold value of € 20,000 per DALY (Fig. 2). Pre- and post-law helmet-wearing rates barely influenced the results. Figure 3 shows that decreasing the annual helmet costs to 2/3rd of the standard helmet costs would also lead to cost-effectiveness for the age groups 0–14 and 15–64 years.

## Discussion

The current study aimed to assess the cost-effectiveness of enforcing bicycle helmet use through legislation as a means of preventing bicycle related TBI and mortality in the Netherlands [49, 50]. Without considering different age groups, the current intervention is not cost-effective in light of the Dutch reference value for cost-effectiveness of € 20,000 per QALY [50]. However, the results show a large fluctuation in cost-effectiveness between age groups. It is notable that all ICERs are most favorable in people aged 65 years or older, despite the fact that this group incurred no costs for lost productivity due to retirement. The absence of savings related to productivity losses was offset by a substantially higher incidence rate and higher mean medical costs when compared to the other groups (Table 2 and Appendix 3). This age group is the only group with an ICER per DALY averted that is below the threshold of € 20,000. In addition, the ICER per death prevented is approximately 26 and 11 times lower as for the youngest and middle age group, respectively.

As no Dutch utility figures for injuries associated with traffic accidents were available, we choose to use disability figures for injuries and DALY outcomes in our analysis. Although use of DALY as an outcome measure in economic evaluations is less common than use of QALYs, it is accepted as well, as appeared from a review of Oostvogels et al. [51]. Therefore, the intended health effect is expressed as DALYs averted following mandatory helmet use. Both QALY and DALY are a form of health-adjusted life expectancy (HALE). The primary difference between the two outcome measures is that QALYs primarily measure health gains following interventions, while DALYs measure health losses from disease and death [52]. Another difference is that DALYs, by adjusting life years for disability caused by one single health problem such as TBI, do not consider comorbidity and thus tend to be relatively larger than QALYs when comorbidity is present. Therefore, use of DALYs could lead to an overestimation of the disease burden [52] and a somewhat flattered ICER. In absence of a clear threshold value in the Netherlands for cost-effectiveness when DALYs are used as outcome measure, we chose to use the same threshold value for DALYs as is commonly used for QALYs in the Netherlands, namely € 20,000 [50]. This threshold value is much more stringent than the WHO guidelines on cost-effectiveness that state that any intervention with a cost-effectiveness ratio below the GDP per capita (€ 43,100 for the Netherlands in 2017) should be regarded as a cost-effective intervention. Should we have followed this standard, our conclusions on cost-effectiveness of mandatory helmet use would have been more positive than they currently are.

When interpreting the results, it should be remembered that we chose not to include the often cited substitution effect and risk compensation effect in our analysis [53, 54] . The former is related to the fact that mandatory helmet use could discourage current cyclists to use bicycles but other forms of transportation instead for (part of) their journeys [53, 54]. The latter is said to increase risk for injury by increased risk taking behavior by either the helmet wearing cyclists themselves or by other traffic participants [55, 56]. However, both mechanisms are not undisputed either [57]. We decided not to include these factors in our model, due to their uncertain nature. Nonetheless, we should bear in mind that the possible presence of substitution and risk compensation effects could have led to less favorable cost-effectiveness estimates.

Our findings are in line with the existing literature. However, most previous economic evaluations of helmet laws have used cost-benefit methods rather than cost-effectiveness analysis [36, 43]. Economic evaluations that use a societal perspective of costs generally agree that a helmet law is not beneficial, i.e. that the costs outweigh the associated benefits [43]. Although many studies evaluate the effectiveness of bicycle helmet laws, not many evaluate their cost-effectiveness. To our knowledge, no contribution has been made to this field in the last two decades. In one of the few papers on cost-effectiveness, Hendrie et al. focused on the cost of public education campaigns and police enforcement and not on medical costs and productivity losses [38]. They did however measure the cost of purchasing helmets, which was also by far their largest cost item. They estimated the cost per TBI prevented to be € 99,123 for their aggregated data model and € 212,769 for their individual pooled data model [38]. This is substantially higher than the €31,028 per TBI prevented that we found, probably mainly because they did not consider the healthcare savings associated with less TBI. In another cost-effectiveness paper, cost per (discounted) life saved was estimated to between € 85,727 to € 110,332 for children from 5 to 12 years, between € 673,193 to € 793,338 for children from 13 to 18 years old and between € 863,340 to € 984,404 for adults [58]. This is substantially lower than the estimates that we found. Interestingly, the cost per death averted in this study is higher for adults than it is for children, while we found an inverse relationship. The most recent cost-effectiveness research was done in 2000 by Kopjar & Wickizer, and found the same age gradient [59]. They found the risk of head injury and the largest reduction in absolute risk of head injury due to wearing a helmet to be the highest in children. We found the opposite. Our data clearly show that, in the Netherlands, 0.115% of all people older than 65 years had TBI in 2017, while only 0.050% of children under 15 years was admitted to an Emergency Department with this type of trauma. This might be because the cycling infrastructure in the Netherlands gives this age group a feeling of safety, while they are in fact more vulnerable [60]. To our knowledge, no other research has looked at the cost per DALY averted or QALY gained to date.

Results of the sensitivity analyses show that the ICERs were most influenced by the efficacy of bicycle helmet use that was assumed in the model. The efficacy of bicycle helmets is often debated in the scientific literature [18, 61–63]. Generally, two types of research have been performed. Firstly, case-control studies, in which brain damage between people that did and did not wear a helmet is compared, and secondly, ecological studies, in which the period before and after the introduction of an intervention to stimulate helmet use is compared. Case-control studies tend to find a higher efficacy of bicycle helmets than time series analysis [64]. Both types of research are vulnerable to confounding factors in their own regard. The meta-analysis by Olivier et al. that was used in this study includes recent research from several countries over several years and was therefore the best available study [18]. Additionally, they checked for evidence of time trends and publication bias, which they did not find. Therefore, this meta-analysis was deemed more comprehensive and likely more fitting the Dutch context, as opposed to results from ecological studies, which generally relate to developments over time in one specific country.

In the Netherlands, the share of electric bicycles out of all newly sold bikes rose from 15% in 2011 to 31% in 2017 [65]. According to the Dutch cyclists’ federation, about 6% of all bicycles in the Netherlands are electric bicycles [66]. However, more than a quarter of all bicycle related deaths were related to use of electric bicycles. Out of these deaths, three quarters are people aged 65 years and older [67]. The relatively high mortality under users of electric bicycles hints to a high incidence of TBI in this group. Therefore, users of electric bicycles seem to be a very relevant group for the intervention under study. Unfortunately, the EDs in the Netherlands do not systematically register the use of an electric bicycle by patients admitted with TBI. Hence, we could not stratify for the use of electric bicycles in our research, while we know that between 2010 and 2017 there was an increase of the selling of new e-bikes of 77%.

### Strengths of this study

The main strength of the study lies in the use of recent data regarding incidence, medical costs, and disease burden from the Dutch Injury Surveillance System and Dutch Burden of Injury Model. Therefore, to our knowledge, this is the first paper that reports the disease burden of bicycle related TBI in DALYs and the societal costs associated with preventing them.

### Limitations of this study

The availability of data used in this research restricted us from performing probabilistic sensitivity analysis (PSA). PSA would have resulted in more reliable and specific ICERs by adding information about the distribution of the model parameters. However, information about the distribution was unavailable for a large number of parameters. The probability of TBI is unrelated to the intensity of bicycle use, while those who cycle more are at greater exposure to TBI risk than those who rarely use a bike. No data are available about bike use intensity. Unfortunately, data on electric bicycles use were not available so we could not distinguish this group of cyclists in this economic evaluation. The fact that we only had healthcare costs available for the first year leads to an underestimation of the cost-effectiveness of the use. Finally, we did not include police enforcement and regulation costs in our study, due to data limitations. The ICERs as estimated in this study may be less beneficial when these costs would have been taken into account. On the other hand, the fact that we only included TBI and not all other injury costs, such as related to fractures, nor other costs of accidents, such as material damage and indirect costs of traffic jams, may have prevented us from reporting more favorable ICERs.

Our results are specific for the Dutch cycling context and, as a consequence, not directly transferable to other countries’ settings. First, in the Netherlands cycling is much more common than in other European countries, Denmark excluded. The infrastructure with cycle lanes and other traffic aspects, such as right of way for cyclists and legal responsibility of motorized traffic in any traffic accident, regardless of actual responsibility, differs enormously from other countries. Consequently, injury risks differ substantially, independent of helmet use. Second, in the Netherlands most cyclist currently do not wear a helmet, whereas in most other countries helmet use is common.

## Conclusions

The current research shows that a law that enforces the use of bicycle helmets for every age group is not cost-effective with an ICER of €28,465 per DALY averted which is higher than the accepted Dutch reference value for cost-effectiveness of preventive interventions. With respect to the difference between age groups, we found this intervention to be cost-effective for people aged 65 years and over due to their relatively high risk of getting TBI with an ICER of €17,775 per DALY averted. One must realize that the costs of the intervention are primarily paid by the individual citizen and the revenues are mainly for the whole society. The acquisition of helmets proved to be an important cost item. If the price of a helmet reduces with 20%, which is a possibility due to large-scale purchases and use of these helmets, the introduction of this regulation would result in an intervention that is approximately cost-effective for all age groups. As this is the first study in the Netherlands that evaluates the cost-effectiveness of enforcing helmet use for cyclists, this can add to the debate around regulating helmet use in general and more specifically for the elderly. We recommend future research towards the mechanisms behind the increased cycling risks for the elderly and the acceptability of a helmet law. We also advise to make the registration of the use of an electric bicycle at the ED standard practice, as the electric bicycle is likely to have an increasing and significant role in TBI. To conclude, although Dutch society is at present not very enthusiastic to wear bicycle helmets, a law that enforces helmet use may be an effective and also cost-effective intervention, certainly in the elderly.

## Data Availability

All data and materials from Statistics Netherlands, the National Road Traffic Database and the Dutch Injury Surveillance System used in the model are freely available by contacting the corresponding author: paul.van.gils@rivm.nl

## Appendix 1

**Table 4:** Number of cyclists by age group

Age	N
− 14	2,337,344
15–64	9,036,764
65+	2,094,634
Total	13,468,742

## Appendix 2

**Table 5:** Overview of incremental cost-effectiveness ratios, human-capital method

Age	Cost per TBI	Cost per death	Cost per DALY
−14	€ 45,158	€ 9,727,999	€ 27,534
15–64	€ 30,738	€ 3,789,708	€ 27,430
65+	€ 14,065	€ 410,871	€ 17,775
Total	€ 27,630	€ 1,783,422	€ 25,348

## Appendix 3

**Table 6:** Mean healthcare costs

Age	Healthcare costs
−14	€ 45,158
15–64	€ 30,738
65+	€ 14,065
Total	€ 27,630

## Abbreviations

DALY: Disability adjusted life year
ED: Emergency department
ICER: Incremental cost-effectiveness ratio
LIS: Injury surveillance system
OR: Odds ratio
PSA: Probabilistic sensitivity analysis
RR: Relative risk
TBI: Traumatic brain injury
YLD: Life years lived with a disability
YLL: Years of life lost
